# New Copper(II)-L-Dipeptide-Bathophenanthroline Complexes as Potential Anticancer Agents—Synthesis, Characterization and Cytotoxicity Studies—And Comparative DNA-Binding Study of Related Phen Complexes

**DOI:** 10.3390/molecules28020896

**Published:** 2023-01-16

**Authors:** Carlos Y. Fernández, Natalia Alvarez, Analu Rocha, Javier Ellena, Antonio J. Costa-Filho, Alzir A. Batista, Gianella Facchin

**Affiliations:** 1 Facultad de Química, Universidad de la República, Av. General Flores 2124, CC1157, Montevideo 11800, Uruguay; 2 Programa de Posgrados de la Facultad de Química, Universidad de la República, Av. General Flores 2124, Montevideo 11800, Uruguay; 3 Departamento de Química, Universidade Federal de São Carlos, CP 676, São Carlos 13565-905, SP, Brazil; 4 Instituto de Física de São Carlos, Universidade de São Paulo, Av. do Trabalhador São-Carlense 400, São Carlos 143566-590, SP, Brazil; 5 Faculdade de Filosofia, Ciências e Letras de Ribeirão Preto, Universidade de São Paulo, Av. Bandeirantes, Ribeirão Preto 14040-901, SP, Brazil

**Keywords:** copper complexes, dipeptide, 4,7-diphenyl-1,10-phenanthroline, DNA interaction, cytotoxic activity

## Abstract

Searching for new copper compounds which may be useful as antitumor drugs, a series of new [Cu(L-dipeptide)(batho)] (batho:4,7-diphenyl-1,10-phenanthroline, L-dipeptide: Gly-Val, Gly-Phe, Ala-Gly, Ala-Ala, Ala-Phe, Phe-Ala, Phe-Val and Phe-Phe) complexes were synthesized and characterized. To interpret the experimental IR spectra, [Cu(ala-gly)(batho)] was modelled in the gas phase using DFT at the B3LYP/LANL2DZ level of theory and the calculated vibrational frequencies were analyzed. Solid-state characterization is in agreement with pentacoordinate complexes of the general formula [Cu(L-dipeptide)(batho)]·x solvent, similar to other [Cu(L-dipeptide)(diimine)] complexes. In solution, the major species are heteroleptic, as in the solid state. The mode of binding to the DNA was evaluated by different techniques, to understand the role of the diimine and the dipeptide. To this end, studies were also performed with complexes [CuCl_2_(diimine)], [Cu(L-dipeptide)(diimine)] and free diimines, with phenanthroline, neocuproine and 3,4,7,8-tetramethyl-phenanthroline. The cytotoxicity of the complexes was determined on human cancer cell lines MDA-MB-231, MCF-7 (breast, the first triple negative), and A549 (lung epithelial) and non-tumor cell lines MRC-5 (lung) and MCF-10A (breast). [Cu(L-dipeptide)(batho)] complexes are highly cytotoxic as compared to cisplatin and [Cu(L-dipeptide)(phenanthroline)] complexes, being potential candidates to study their in vivo activity in the treatments of aggressive tumors for which there is no curative pharmacological treatment.

## 1. Introduction

Cancer causes a sanitary burden, with approximately 19 million new cancer cases and almost 10 million cancer deaths a year worldwide (estimated data for 2020). Female breast cancer is the most diagnosed cancer. This global cancer burden is expected to rise to more than 28 million cases per year by 2040 [1]. Several anticancer drugs are available, but they fail to achieve the desired therapeutic effect in all patients, and cause severe side effects. Therefore, it is necessary to identify and develop more effective and safe anticancer drugs [2,3].

The development of therapeutic agents may benefit from using metal coordination compounds, to exploit their chemical and structural versatility in synergy with organic ligands. Despite that, the research on coordination compounds as drugs has remained mainly in the academic media, perhaps due to the high variety of reactivity they present, including chemical speciation [4].

The discovery of the antitumor activity of cisplatin, which presents high chances of cure of testicular cancer and aids in the treatment of other classes of cancer, led to the development of other platinum complexes for cancer treatment, and several of them are currently in clinical use. This also incentivized the research on complexes of other metals [5].

The research of copper complexes as antitumor agents started under the hypothesis that, as copper is an essential metal and there are specific metabolic routes for it, its complexes may present fewer side effects than other metals [3]. To date, research on copper complexes is performed taking into account the different mechanism of action and spectrum of activity observed when compared to available drugs [6]. 

This potential of copper complexes to produce antitumor compounds has led to the development of several copper complexes that present antitumor activity, even when the ligands are biologically inactive [3]. The compounds in the family Casiopeinas^®^ are among the most studied copper complexes, with Casiopeina III-ia, [(Cu(II))(4,4′-dimethyl-2,2′-bipyridine)(acetylacetonate)(NO_3_)(H_2_O)], being tested in a clinical phase I trial in Mexico [7,8]. Another relevant compound is HydroCuP^®^, [Cu(tris-hydroxymethylphosphino)_4_][PF_6_], which is highly selective towards cancer cells and has presented promising results in advanced preclinical studies [9,10,11]. Cancer stem cells (CSC) are a subset of tumor cells that can survive traditional cancer treatments and generate a progeny of differentiated cells, leading to cancer relapse. Copper complexes containing bathophenanthroline (batho) are emerging as tools to fight CSCs [12]. It has been demonstrated that a batho complex induces breast CSC immunogenic cell death [13].

The mechanism of action of copper complexes is not completely understood and includes different molecular events. The lack of specificity against a single molecular target strengthens the copper complex’s ability to fight a diverse cell population such as those found in a tumor. It is accepted that most complexes produce ROS, to which tumor cells are especially susceptible. Many complexes bind to the DNA, as determined in vitro, which possibly, combined with ROS production, is among the first molecular events triggered by the complexes [4,14,15,16]. Other mechanisms are also emerging [4,14,15], among the most recent being the so-called Cuproptosis described by Tsvetkov et al. [17].

Our research group has been working to develop new copper compounds with a cytotoxic activity which may lead to anticancer agents. Different series of [Cu(L-dipeptide)(diimine)] compounds were synthesized and characterized (where diimine: phenanthroline, phen, 5-NO_2_-phenanthroline, 5-NO_2_-phen, neocuproine, neo and 3,4,7,8-tetramethyl-phenanthroline, tmp) [18,19,20,21,22]. In general, they are potent cytotoxic agents, more active than cisplatin, with neo and tmp complexes being the most active of the group. We look forward to developing compounds with improved spectra of activity against cancer cells. In this work, we selected batho as a diiminic ligand due to references to batho complexes with high activity against cancer cells, including CSCs [12], trying to merge its activity with that of the Cu-dipeptide complexes, which are also a very stable scaffold to bind the diimine [23]. The set of L-dipeptides (L-dipeptides: Gly-Gly, Gly-Phe, Ala-Gly, Ala-Ala, Ala-Phe, Val-Phe, Phe-Ala and Phe-Phe) was selected to cover a range of different side chains and lipophilicity. The complexes were characterized both in the solid state and in aqueous solution. The complex [Cu(ala-gly)(batho)] was modelled in the gas phase using DFT, and the calculated vibrational frequencies were analyzed in order to interpret the experimental IR spectra. The binding of the complexes to the DNA was studied by UV, determining *K_b_*, and viscosity methods. To evaluate the effect of the phen substituents in the DNA-binding characteristics, related complexes of phen, neo, and tmp were also included in this study. Finally, the cytotoxicity of the complexes was evaluated against MDA-MB-231, MCF-7 (human metastatic breast adenocarcinomas, the first triple negative), MCF-10A (human non-tumor breast cells), A549 (human lung epithelial carcinoma) and MRC-5 (human non-tumor lung epithelial cells). 

## 2. Results and Discussion

**Figure 1:**
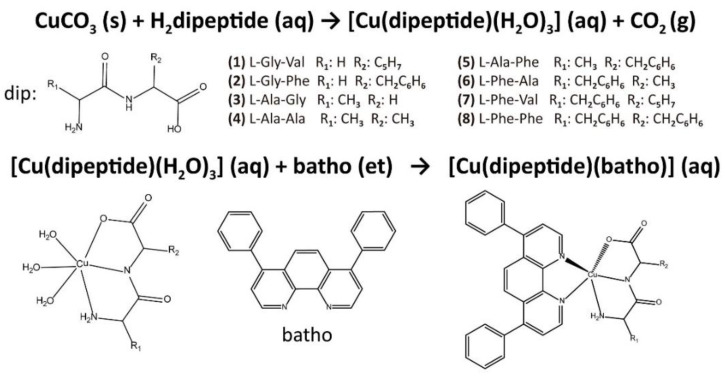
Scheme of the proposed structure of complexes and synthetic scheme.

As described in the experimental section, eight new complexes—[Cu(L-Gly-Val)(batho)]·CH_3_CH_2_OH·3H_2_O, **1**; [Cu(L-Gly-Phe)(batho)]·CH_3_CH_2_OH·5.5H_2_O, **2**; [Cu(L-Ala-Gly)(batho)]·3H_2_O, **3**; [Cu(L-Ala-Ala)(batho)]·CH_3_CH_2_OH·4H_2_O, **4**; [Cu(L-Ala-Phe)(batho)]·CH_3_CH_2_OH·4.5H_2_O, **5**; [Cu(L-Phe-Ala)(batho)]·1.5CH_3_CH_2_OH·3H_2_O, **6**; [Cu(L-Phe-Val)(batho)]·0.5CH_3_CH_2_OH·10H_2_O, **7**; and [Cu(L-Phe-Phe)(batho)]·3CH_3_CH_2_OH·9H_2_O, **8**—were obtained according to the scheme in Figure 1. When necessary, comparative studies were performed with other complexes.

### 2.1. Geometry Optimization, IR Spectrum Calculation, and Interpretation

**Figure 2:**
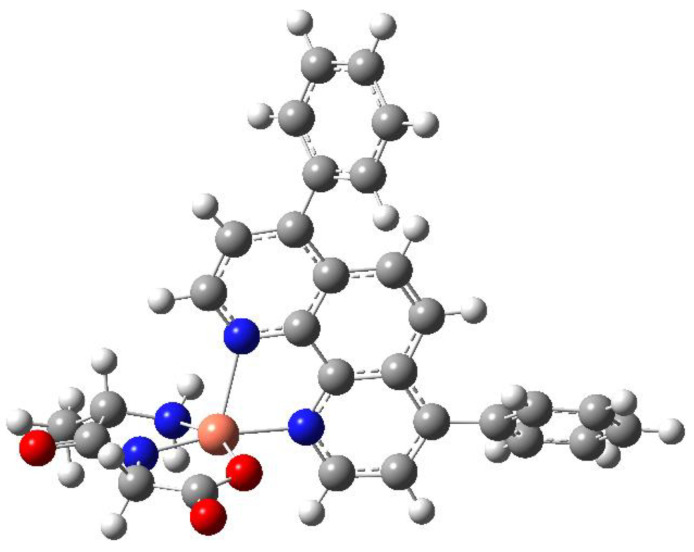
Optimized geometry for compound **3**.

To better understand the structure of the complexes and taking into account the determined structure of related complexes, the optimized geometry for [Cu(L-Ala-Gly)(batho)], corresponding to compound **3,** was calculated, confirming it referred to an energy minimum. It presents a pentacoordinate copper(II) center with a N3O equatorial coordination, where 2 N and 1 O atoms come from the dipeptide ligand, whereas the third N atom comes from the batho ligand, which is perpendicular to the plane defined by the dipeptide. The coordination and spatial arrangement are similar to those in the crystal structures of previously reported [Cu(dipeptide)(diimine)] complexes where diimine is: phen [18], 5-NO_2_-phen [19], neo [20] and tmp [22]. Figure 2 presents the optimized geometry. (Figure S1, Supplementary Materials, presents a space fill representation of it.).

The vibrational spectrum for compound **3** was calculated, and vibrational modes were assigned based on potential energy distribution (PED) information. The assignment and corresponding frequencies of selected vibrational modes are shown in Table 1. For clarity purposes, the vibrational modes for bonds are discriminated by ligand (batho and dipeptide).

Despite being performed in the gas phase, calculations led to a good agreement between experimental and calculated values accounting for the validity of the computational model. The differences between the calculated and experimental frequencies regarding the N-H stretching, in which the experimental frequencies are lower, are possibly due to the participation of these bonds in intermolecular H bonding in the solid state that is unaccounted for in the gas phase model. 

### 2.2. Solid-State Characterization: Infrared Spectra

All the studied heteroleptic complexes present similar infrared spectra. IR spectra of the obtained complexes were assigned taking into account the calculated IR and interpretation for compound **3**, as well as the previously reported data for related complexes [18,20,22,23,24,25,26]. The common characteristic bands in the spectra (Table 2), include a broad, very strong peak at approximately 1600 cm^−1^ corresponding to ν(C=O) + ν(C-N) + ν_as_(COO), which is characteristic of the coordinated dipeptide moiety. This broad peak is superimposed on the one assigned to the batho ring ν(C-C) stretching. Absorption peaks corresponding to other ring stretching frequencies of the batho are modified in relation to the free ligand and very close to those of the [Cu_2_Cl_4_(batho)_2_]·H_2_O (Cu-batho) appearing at approximately 1530 and 1400 cm^−1^, in agreement with the coordination of batho. 

**Figure 3:**
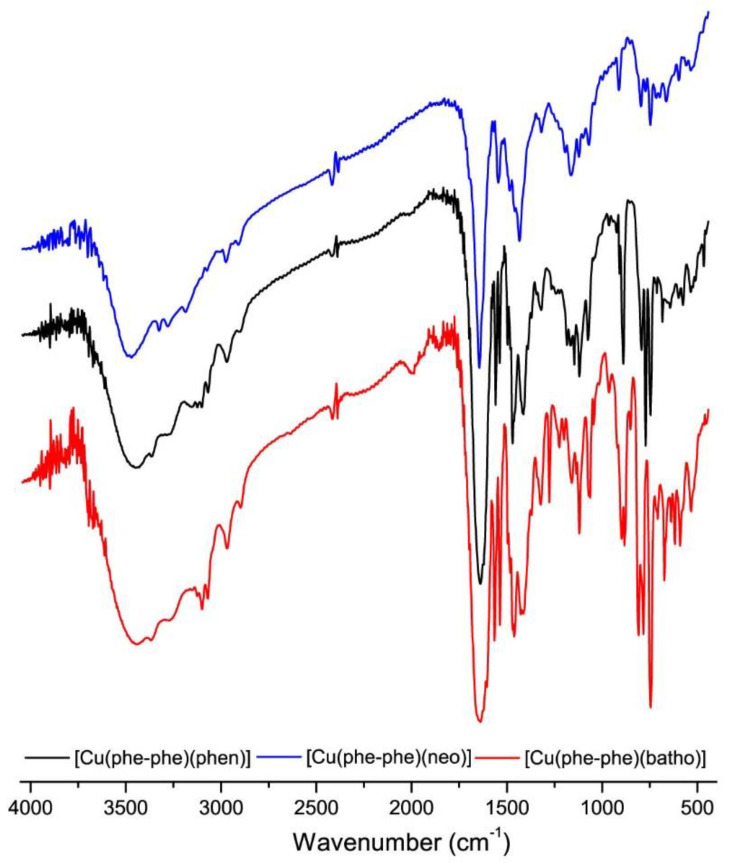
Superimposed IR spectra of: [Cu(L-Phe-phe)(diimine)] with diimine: phen (black), neo (blue), and batho (red).

The obtained IR spectra of the complexes are very similar to those of the corresponding [Cu(dipeptide)(diimine)] complexes whose crystal structures have been determined. For instance, Figure 3 shows the superposition of the spectra of [Cu(phe-phe)(diimine)] with diimine phen, neo, and batho complexes. This supports the hypothesis that the coordination in all these families of complexes is the same, with relatively similar structures, as proposed in Figure 1, in agreement with the optimized structure of **3**.

### 2.3. Characterization in Solution UV–Visible Spectra

To gain insight into the major species in solution, electronic spectra of the complexes were recorded and analyzed. All complexes present a broad peak at approximately 610 nm with a shoulder at approximately 850 nm. This is typical of Cu(II) in pentacoordinate complexes. The wavelength of the maximum absorption (λ_max_) and absorptivity values are listed in Table 3. 

An empirical correlation between the visible spectrum λ_max_ and the donor atoms coordinated to the Cu(II) was used to analyze the experimental spectra [27,28]. The λ_max_ of the visible spectra, calculated according to *Prenesti* et al. [28,29] for the coordination scheme shown in Figure 1 (corresponding to proposed solid-state coordination) is approximately 610–620 nm, similar to the experimental value in DMSO. This suggests that the pentacoordinate heteroleptic complex is the solution’s major species, not excluding others’ existence, as observed for [Cu(L-dipeptide)(phen)], [Cu(L-dipeptide)(neo)], and [Cu(L-dipeptide)(tmp)] complexes [18,20,22]. In a DMSO:water (50:50) solution, the same spectral characteristics were observed, with λ_max_ shifted to 620–630 nm. 

There are no significant changes in spectra during 48 h (both in DMSO and DMSO:water). Therefore, complexes are stable during this period in solution. Conductivity measurements also are stable with time (values in the 0–2 µS at 1 mM in DMSO).

### 2.4. DNA binding

Values of intrinsic binding constants to DNA (*K_b_*) of the homoleptic and heteroleptic complexes were similar, with values of approximately 1 × 10^3^ (Table S1, Supplementary Materials). The *K_b_* values are approximately ten-fold lower than those of the corresponding [Cu(L-dipeptide)(phen)] [18] and similar to those of [Cu(L-dipeptide)(tmp)] [22] complexes, suggesting that phenyl groups impair DNA binding as compared to phen, similarly to methyl groups of tmp. 

The viscosity of the DNA is highly sensitive to changes in the DNA’s length. Its study is considered among the most reliable techniques for DNA binding mode analysis in solution. DNA base pairs tend to separate to accommodate an intercalated molecule into the helix, increasing the length of the DNA, leading to a viscosity increase. Other binding modes exert minor modifications on DNA viscosity [30,31].

**Figure 4:**
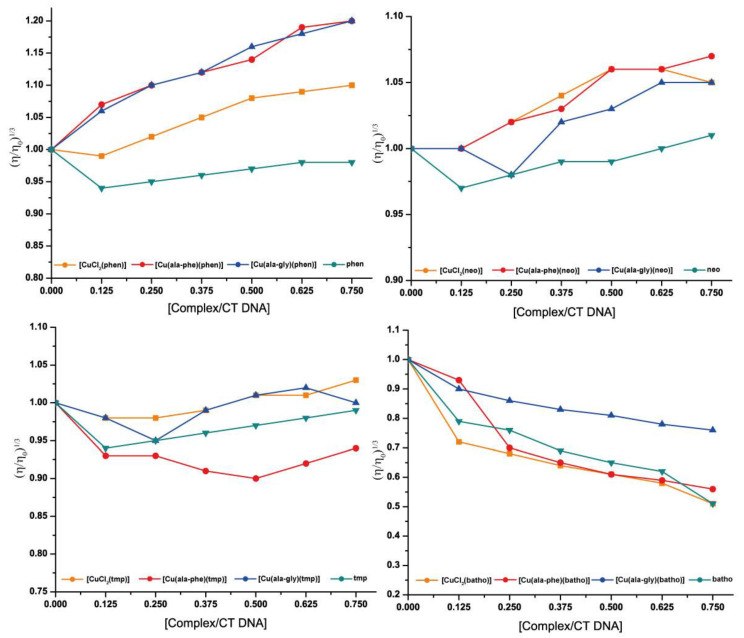
Effect of the increasing concentration of complexes on the relative viscosity of CT -DNA. [DNA] = 150 μM, for the free diimine, Cu-diimine and [Cu(L-dipeptide)(diimine)] for diimine: phen, neo tmp and batho.

The relative viscosity of CT-DNA in the presence of compounds **3** and **5**, the homoleptic Cu-batho and free batho compounds as well as the related complexes of phen, tmp and neo were determined. Figure 4 presents the obtained results. 

In relation to the free diimine DNA binding, it is observed that the relative viscosity decreases at the complex/DNA ratio of 0.125. Such behavior may be explained by a binding mode that produces bends or kinks in the DNA helix, as observed for some partial or non-classical intercalators, including the Δ-[Ru(phen)_3_]^2+^ complex [32,33]. At higher complex/DNA ratios for phen, neo and tmp it slightly increases, whereas for batho it continues to decrease. This observation cannot be straightforwardly explained. It can be hypothesized that at low complex/DNA ratios, the diimne binding induces the DNA to bend. The positions for that mode of binding are saturated at ratios higher than 0.125 for phen, neo and tmp and when the diimine is present at higher ratios, it binds in a different mode. 

Phen-containing complexes augmented DNA relative viscosity, with Cu-phen inducing a significant increase. The Cu-phen plot slope (0.27) is near the estimated one for a partial or non-classical intercalator [34] (the slope for ethidium bromide, a classical intercalator, is approximately 1 [31]). This agrees with the partial intercalation and groove binding to DNA detected for this complex via fiber EPR studies [35]. The heteroleptic complexes induced a similar increase in viscosity. A similar behavior was found for phen-containing complexes Casiopeinas, where both intercalation and minor groove binding are present, to different extents depending on the anionic ligand [36]. Experiments were repeated in water without using DMSO, yielding the same results. Neo and neo-containing complexes present a less marked increase in viscosity than phen complexes, possibly accounting only for groove binding (slope approximately 0.1), as detected for Cu-tmp complex by fiber EPR studies [35]. Tmp complexes induce no significant alteration of DNA viscosity. This pattern of methyl groups impairing DNA intercalation agrees with that reported by Palaniandavar et al. [37] for related [Cu(diimine)_2_]^2+^ complexes.

Free batho and batho-containing complexes displayed a markedly different behavior. Viscosity decreased in the presence of the compounds, with a negative slope of approximately 0.3 (approximate slopes are included in Table S2, Supplementary Materials). This may result from groove binding inducing bends on the DNA (since the phenomenon is also observed for free batho covalent binding is discarded) possibly by partial intercalation of the phenyl groups of batho [31].

To sum up, phen complexes studied in this work, both homoleptic and heteroleptic, may intercalate to DNA as well as binding in the grooves. Neo and possibly tmp complexes, homoleptic and heteroleptic, do not evidence intercalation, possibly binding in the grooves. For the batho complexes (and free batho), a different pattern of viscosity changes was observed that suggests that batho binding induces bends in the DNA possibly as a result of partial intercalation of the phenyl groups.

### 2.5. Cytotoxicity

The complexes were highly cytotoxic against the studied cell lines, as presented in Table 4. Most complexes showed much higher activity than cisplatin on the studied cancer cells. 

Compared with other Cu compounds, the complexes can be classified as potent or remarkable cytotoxic agents according to the classification of Santini et al. [3] as they present IC_50_ in the low µM range. In general, complexes are highly active if compared with other heteroleptic complexes containing a phen based ligand [15], including Casiopeínas [7]. As compared with others [Cu(dipeptide)(diimines)] compounds, the cytotoxicity depends more on the diimine than on the dipeptide, with the activity increasing in the order phen ≅ 5-NO_2_-phen < batho <= neo <= tmp. Despite not being the more active compounds, batho complexes are in general slightly more selective than the other [Cu(L-dipeptide)(diimine)] complexes (Table S3, Supplementary Materials). Therefore, [Cu(dipeptide)(batho)] complexes are interesting compounds to further study their biological activity especially, their anti-breast CSC activity, for instance [Cu(gly-val)(batho)] and [CuCl_2_(batho)] could be tested on triple negative breast cancer.

## 3. Materials and Methods

All reagents for the synthesis and biochemical studies were used as purchased without further purification: copper salts (Fluka), L-dipeptides (SIGMA, Sigma-Aldrich, St. Louis, MO, USA), bathophenanthroline (4,7-diphenyl-1,10-phenanthroline, SIGMA) and calf thymus-DNA (CT-DNA, SIGMA).

### 3.1. Synthesis and Analytical Characterization

Firstly, the [Cu(dipeptide)] precursor was obtained by dissolving the dipeptide in the minimum volume of water. To this solution, a 50% excess of CuCO_3_ (in relation to the dipeptide) was added and stirred at 60–80 °C for 1 h. After that, the remaining excess of CuCO_3_ was filtered off. The resulting blue solution was evaporated at 60–80 °C until an adequate amount of solid is obtained which was then filtered, washed with cold water and air dried [24,25,38]. The L-dipeptides were: Gly-Val, Gly-Phe, Ala-Gly, Ala-Ala, Ala-Phe, Val-Phe, Phe-Ala and Phe-Phe. Equimolar amounts of [Cu(dipeptide)] in a water solution and batho in an ethanolic solution were mixed while constant stirring for 15 min at 60 °C. Amorphous solid was obtained after solvent evaporation at room temperature, with yields ranging from 50 to 70%. Several attempts were unsuccessful in obtaining single crystals by varying the temperature and solvent mixtures.

Elemental analysis for C, N, H and S was performed in a Thermo Flash 2000 equipment and results are as follows: [Cu(L-Gly-Val)(batho)]·CH_3_CH_2_OH·3H_2_O, **1,** Calc./Found (CuC_33_N_4_H_40_O_7_) %C: 59.31/59.62, %N: 8.38/8.26, %H: 6.03/5.62; [Cu(L-Gly-Phe)(batho)]·CH_3_CH_2_OH·5.5H_2_O, **2,** Calc./Found (CuC_37_N_4_H_45_O_9.5_) %C: 58.37/58.16, %N: 7.36/7.39, %H: 5.95/5.53; [Cu(L-Ala-Gly)(batho)]·3H_2_O, **3,** Calc./Found (CuC_31_N_4_H_34_O_6_) %C: 59.44/60.19, %N: 9.01/9.05, %H: 4.98/5.08; [Cu(L-Ala-Ala)(batho)]·CH_3_CH_2_OH·4H_2_O, **4,** Calc./Found (CuC_32_N_4_H_40_O_8_) %C: 57.17/56.98, %N: 8.33/8.44, %H: 6.00/5.78; [Cu(L-Ala-Phe)(batho)]·CH_3_CH_2_OH·4.5H_2_O, **5,** Calc./Found (CuC_38_N_4_H_45_O_8.5_) %C: 60.27/60.54, %N: 7.40/7.37, %H: 5.99/5.67; [Cu(L-Phe-Ala)(batho)]·1.5CH_3_CH_2_OH·3H_2_O, **6,** Calc./Found (CuC_39_N_4_H_45_O_7.5_) %C: 62.18/62.30, %N: 7.44/7.55, %H: 6.02/5.69; [Cu(L-Phe-Val)(batho)]·0.5CH_3_CH_2_OH·10H_2_O, **7,** Calc./Found (CuC_40_N_4_H_60_O_14_) %C: 54.44/54.51, %N: 6.68/6.60, %H: 6.59/6.22; [Cu(L-Phe-Phe)(batho)]·3CH_3_CH_2_OH·9H_2_O, and **C8** and Calc./Found (CuC_47_N_4_H_66_O_14_) %C: 57.27/57.95, %N: 5.56/5.78, %H: 7.00/6.59.

### 3.2. DFT Studies (Geometry Optimization and Infrared Spectra)

The proposed starting geometry for the pentacoordinate compound **3** was based on the crystal structure of the previously reported phen analogue [18] and modified in Gaussview 5.0 [39]. Geometry optimization in the gas phase was performed using the density functional theory method (DFT) [40] with the B3LYP functional [41] and the LANL2DZ basis set [42,43,44]. Calculations were performed on Gaussian 09 software [45]. Upon completion, all determined frequencies presented real values confirming it referred to an energy minimum. 

Potential energy distribution (PED) analysis was performed on the calculated infrared spectra using VEDA software [46]. 

### 3.3. Spectroscopic Characterization

Infrared spectra of the compounds in KBr pellets were recorded on a Shimadzu IR Prestige 21 spectrometer in the 4000 to 400 cm^−1^ range using 20 accumulations and a resolution of 4 cm^−1^.

Solution electronic (UV–vis) spectra of the complexes were carried out in a Thermo Scientific Evolution 60 spectrophotometer, using 1 cm path length quartz cells, in 5 mM DMSO solutions and in 2.5 DMSO: H_2_O 50:50 (complexes are not soluble in pure H_2_O). 

### 3.4. DNA Interaction

#### 3.4.1. Determination of *K_b_ via* the UV Absorption Titration Experiments

Absorption titration measurements were carried out keeping the complex concentration constant at 10–15 μM in 5 mM buffer Tris/HCl pH = 7.5 and 50 mM of NaCl while varying the concentration of calf thymus-DNA (CT-DNA) from 0 to 250 μM. The intrinsic binding constants (*K_b_*) were determined using the Benesi–Hildebrand method [47], by calculating the ratio of the slope to the intercept of the [complex]/A_obs_ as a function of 1/[DNA] plot.

#### 3.4.2. Viscosity Studies

Measurements of viscosity were performed in an Ostwald-type viscosimeter maintained at a constant temperature of 25.0 ± 0.1 °C in a thermostatic bath.

Solutions of calf thymus-DNA (CT-DNA, 150 μM b.p.) and compounds were prepared, separately in Tris-HCl (10 mM, pH = 7.2) and thermostatized at 25 °C. Complex−DNA solutions were prepared just prior to running each experiment, (6 mL) at different molar ratios ([complex]/[CT-DNA] = 0.125, 0.250, 0.375, 0.500, 0.625 and 0.750). Solutions were equilibrated for 15 min at 25 °C and then 5 flow times were registered.

The relative viscosity of DNA in the absence (η_0_) and presence (η) of complexes was calculated as: (η/η_0_) = t − t_0_/t_DNA_ − t_0_, where t_0_ and t_DNA_ are the flow times of the buffer and DNA solution alone, respectively, while t is the flow time of the DNA solution in the presence of copper compounds. Data are presented as (η/η0)^1/3^ versus the ratio [complex]/[DNA] [48]. 

### 3.5. Cytotoxicity Studies

The cytotoxicity of the complexes was evaluated against different human cancer cell lines: human metastatic breast adenocarcinoma MDA-MB-231 (triple negative, ATCC: HTB-26), MCF-7 (hormone-dependent ATCC: HTB-22), human lung epithelial carcinoma A549 (ATCC: CCL-185) and non-tumor cell lines MRC-5 (lung; ATCC: CCL-171) and MCF-10A (breast, ATCC: CRL-10317), using the 3-(4,5-dimethylthiazol-2-yl)-2,5-diphenyltetrazolium bromide (MTT) colorimetric assay. The cells were cultured in Dulbecco’s Modified Eagle’s Medium (DMEM) for MDA-MB-231, A549 and MRC-5, supplemented with 10% fetal bovine serum (FBS), Roswell Park Memorial Institute (RPMI) 1640 Medium for MCF-7, supplemented with 10% FBS or Dulbecco’s Modified Eagle Medium Nutrient Mixture F-12 (DMEM F-12) for MCF-10A, containing 5% horse serum, Epidermal growth factor (EGF, 20 ng mL^−1^), hydrocortisone (0.5 μg mL^−1^), insulin (0.01 mg mL^−1^), 1% penicillin and 1% streptomycin, at 310 K in humidified 5% CO_2_ atmosphere. To conduct the assay, 1.5 × 10^4^ cells/well were seeded in 150 μL of medium in 96-well plates and incubated at 310 K in 5% CO_2_ for 24 h to allow cell adhesion. Then, the cells were treated with copper complexes for 48 h. Cu complexes were dissolved in DMSO, and 0.75 μL of solution was added to each well with 150 μL of medium (final concentration of 0.5% DMSO/well). Cisplatin, used as a reference drug, was solubilized in DMF. After the treatment, MTT (50 μL, 1 mg mL^−1^ in PBS) was added to each well, and the plate was incubated for 3 h. Cell viability was detected by the reduction of MTT to purple formazan by living cells. The formazan crystals were solubilized by isopropanol (150 μL/well), and the optical density of each well was measured using a microplate spectrophotometer at a wavelength of 540 nm. The concentration to 50% (IC_50_) of cell viability (Table 4) was obtained from the analysis of absorbance data of three independent experiments.

## 4. Conclusions

Eight new heteroleptic [Cu(L-dipeptide)(batho)] complexes were synthesized and characterized both in the solid state and in solution. The coordination environment of the metal in the solid state is maintained in the major species in solution and is the same as in other [Cu(L-dipeptide)(diimine)] compounds. 

The complexes are highly cytotoxic as compared with other Cu complexes and cisplatin, and are interesting candidates to further study their anti-CSC activity and their in vivo activity.

Batho impairs DNA binding as compared to phen complexes, possibly favoring (major) groove binding, with the dipeptide only modulating the strength of the binding. In spite of that, the introduction of batho as a ligand augmented the cytotoxic activity of the complexes, as compared to the [Cu(L-dipeptide)(phen)], suggesting that the DNA intercalation is not determinant in the cytotoxicity of the compounds.

## Figures and Tables

**Table 1:** Experimental band assignment using PED for **3**. Experimental and calculated frequencies are expressed in cm^−1^.

Experimental	Calculated	PED%	Assignment
3412	3583	99	ν_as_(N-H)
	3477	99	ν_s_(N-H)
3240	3252	98	ν_s_, batho(C-H)
	3244	94	
	3238	80	
	3236	82	
	3235	82	
	3231	94	ν_as_, batho(C-H)
	3221	81	
	3220	61	
	3220	85	
3141	3212	79	ν_as_,batho(C-H)
	3211	93	
	3210	82	
	3200	85	
	3199	83	
	3195	84	
	3194	88	
	3163	84	ν_as_, dipeptide(C-H)
	3117	93	
2917	3083	99	ν_as_, dipeptide(C-H)
	3068	94	
	3038	99	ν_s_, dipeptide(C-H)
	3033	84	
1597	1683	77	δ(H-N-H) dipeptide
	1662	44	ν(C-C) batho
	1653	52	
	1652	53	
	1642	80	ν_as_(COO) dipeptide
1564	1621	81	ν(N-C) + ν(O-C) dipeptide
	1593	32	ν(N-C) batho
1521	1526	49	δ(H-C-C) batho + dipeptide
	1526	64	δ(H-C-H) dipeptide
	1521	51	δ(H-C-C) batho
1492	1508	72	δ(H-C-H) dipeptide
	1499	68	
	1499	29	τ(H-C-C-O) dipeptide
	1475	49	δ(H-C-C) batho
	1472	39	
1427	1433	87	δ(H-C-H) methyl in dipeptide
	1404	46	ν(N-C) + ν(O-C) + ν(C-C) dipeptide
1374	1382	40	τ(H-C-C-N) dipeptide
	1379	46	δ(H-C-C) batho
	1378	29	ν(C-C) batho
	1378	42	δ(H-C-C) batho
	1352	25	ν(C-C) batho
1288	1335	25	δ(H-C-H) dipeptide
	1335	50	τ(H-C-C-O) dipeptide
	1323	55	δ(H-C-C) dipeptide
1232	1248	58	ν_s_(COO) + ν(C-C) dipeptide
	1228	67	δ(H-C-C) batho
	1227	65	
1183	1215	27	δ(H-C-C) batho
	1212	49	δ(H-C-C) dipeptide
	1212	31	τ(H-C-C-O) dipeptide
	1209	78	δ(H-C-C) batho
	1209	78	δ(H-C-C) batho
1157	1130	55	ν(N-C) + ν(C-C) dipeptide
1090	1073	49	ν(N-C) + ν(C-C) dipeptide
1022	1047	28	τ_oop_(H-C-C-C) batho
	1045	62	
	1042	47	
	1025	76	
	1024	27	δ(H-N-C) batho
	1024	34	τ_oop_(H-C-C-N) batho
	1023	73	τ_oop_(H-C-C-C) batho
999	1012	43	τ_oop_(H-C-C-C) batho
	1010	26	δ(H-C-C) dipeptide
	1006	73	τ_oop_(H-C-C-C) batho
	997	53	τ(H-N-C-C) dipeptide + batho
972	970	75	τ_oop_(H-C-C-C) batho
	969	69	
	910	40	δ(C-C-C) + δ(C-C-N) batho
858	903	68	τ_oop_(H-C-C-C) batho
	897	84	
	895	50	τ_oop_(H-C-C-N) batho
	893	25	
	891	65	
	890	64	ν(N-C) + ν(C-C) + ν(O-C) dipeptide
	882	66	τ_oop_(H-C-C-C) batho
843	851	45	ν(N-C) + ν(C-C) + ν(O-C) dipeptide
810	799	38	τ_oop_(H-C-C-C) batho
768	743	48	τ(O-C-N-C) dipeptide
740	732	48	τ_oop_(H-C-C-C) batho
	732	29	τ(C-C-C-C) batho
	731	44	τ_oop_(H-C-C-C) batho
	731	28	τ(C-C-C-C) batho
704	682	61	δ(C-C-O) dipeptide
666	640	39	δ(C-C-C) batho
630	632	28	δ(C-C-C) batho
598	581	47	τ(H-N-C-C) dipeptide
575	564	64	τ(O-C-O-C) dipeptide
548	543	26	δ(C-C-N) dipeptide
521	507	52	δ(C-C-N) + δ(C-C-O) + δ(O-C-O) dipeptide
438	428	37	τ(C-N-C-C) dipeptide
417	421	74	τ(C-C-C-C) batho
	420	72	τ(C-C-C-C) batho

**Table 2:** FTIR spectra assignment for complexes **1**–**8**.

Compound	νs + νas(N-H) *	ν(C=O) * + ν(C-N) ** + ν *_as_* (COO) * + ν(C-C) **	ν(N-C) * + ν(C=O) * + ν(C-C) *	ν_s_(COO) * + ν(C-C) *	ρ(C-H) **	δ(C-C-O)*	δ(C-C-N) * + δ(C-C-O) * + δ(O-C-O) *
**1**	3415sh	1588s, 1516w	1420m	1239w	1040w	704s	536w
**2**	3401sh	1594s, 1516w	1417m	1226w	1040w	704s	542w
**3**	3412sh	1597s, 1521w	1427m	1232w	1094w	704s	575w
**4**	3415sh	1594s, 1516w	1413m	1226w	1084w	704s	549w
**5**	3401sh	1601s, 1523w	1413m	1233w	1065w	704s	542w
**6**	3415sh	1601s, 1516w	1420m	1239w	1064w	704s	549w
**7**	3408sh	1594s, 1516w	1427m	1233m	1065w	704s	536w
**8**	3401sh	1594s, 1523w	1413m	1233m	1072w	704s	555w

* Dipeptide bands; ** batho bands.

**Table 3:** Wavelength of the maximum absorption (λ_max_, nm) and molar absorptivity (ɛ).

Compound	λ_max_ (nm) in DMSO/in Water:DMSO 50:50 *	ɛ (DMSO)
**1**	608	178
**2**	610	147
**3**	615/630	132
**4**	611	92
**5**	610/620	133
**6**	611	142
**7**	610	106
**8**	608	121

* Only for soluble compounds in this condition.

**Table 4:** Cytotoxic activity (expressed by IC_50_ in µM) of the studied complexes after 48 h of incubation, against MCF-7, MDA-MB-231 (human breast adenocarcinomas, the latter triple negative), MCF-10A (breast non-tumor), A549 (human lung epithelial carcinoma), and MRC-5 (lung non-tumor).

	Compound	MDA-MB-231	MCF-7	MCF-10A	A549	MRC-5
**1**	[Cu(gly-val)(batho)]	0.41 ± 0.03	0.40 ± 0.01	4.95 ± 0.94	1.20 ± 0.36	0.60 ± 0.12
**2**	[Cu(gly-phe)(batho)]	1.16 ± 0.22	1.40 ± 0.20	5.28 ± 0.52	1.77 ± 0.22	1.72 ± 0.09
**3**	[Cu(ala-gly)(batho)]	5.30 ± 0.81	1.34 ± 0.80	6.67 ± 1.89	2.18 ± 0.44	0.15 ± 0.03
**4**	[Cu(ala-ala)(batho)]	0.47 ± 0.07	1.45 ± 0.43	3.60 ± 0.31	0.90 ± 0.07	0.61 ± 0.17
**5**	[Cu(ala-phe)(batho)]	0.97 ± 0.20	1.49 ± 0.46	3.86 ± 0.98	0.66 ± 0.20	0.31 ± 0.05
**6**	[Cu(phe-ala)(batho)]	0.79 ± 0.13	0.53 ± 0.09	1.78 ± 0.41	0.33 ± 0.13	0.25 ± 0.02
**7**	[Cu(phe-val)(batho)]	0.65 ± 0.06	1.54 ± 0.96	3.75 ± 0.78	1.47 ± 0.16	0.35 ± 0.05
**8**	[Cu(phe-phe)(batho)]	1.06 ± 0.44	3.34 ± 2.38	4.28 ± 0.75	1.59 ± 0.18	0.99 ± 0.21
**0**	[CuCl_2_(batho)]	0.47 ± 0.07	2.75 ± 0.84	2.75 ± 0.60	0.87 ± 0.11	0.85 ± 0.15
	Cisplatin	12.43 ± 0.20	8.91 ± 2.60	23.90 ± 0.70	14.40 ± 1.40	29.09 ± 0.78

## Data Availability

The data presented in this study are available in the article and supplementary material.

## Supplementary Materials

The following supporting information can be downloaded at: https://www.mdpi.com/article/10.3390/molecules28020896/s1, Figure S1: Space fill representation of the optimized structure of compound 3. Table S1: DNA binding constants (*K_b_*) determined by the Benesi–Hildebrand method. For comparison, previously determined values of related complexes are also included. Table S2: Approximate DNA slope of the variation of the viscosity induced by the binding of the complexes. Table S3: Selectivity index of the compounds (SI, IC_50_ on non-tumor cells/IC_50_ on tumor cells of related origin).
