# Supplementary material for: New Copper(II)-L-Dipeptide-Bathophenanthroline Complexes as Potential Anticancer Agents—Synthesis, Characterization and Cytotoxicity Studies—And Comparative DNA-Binding Study of Related Phen Complexes
Source: Molecules. 2023 Jan 16;28(2):896. doi: 10.3390/molecules28020896 (PMC9863540; doi:10.3390/molecules28020896)
Supplement: Supplementary file 1 [file molecules-28-00896-s001.zip › molecules-2145326-supplementary.pdf]

Supplementary material.

Bathophenanthroline copper complexes as candidates to antitumor drugs. Synthesis, characterization, DNA interaction and cytotoxicity studies of a series of copper(II)-L-dipeptide-bathophenanthroline complexes.

Carlos Y. Fernández[a,b], Natalia Alvarez[a], Analu Rocha[c], Javier Ellena[d], Antonio J. Costa-Filho[e], Alzir A. Batista[c], Gianella Facchin[a*].

Figure S1. Space fill representation of the optimized structure of compound 3.

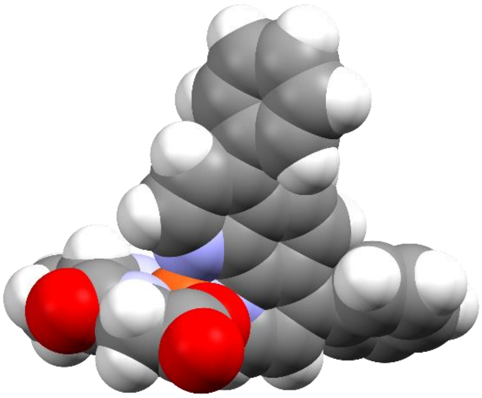

Table S1. DNA binding constants (K_b) determined by the Benesi-Hildebrand method. For comparison previously determined values of related complexes are also included.

Complex	Code	K_b (x10^3 M^{-1})
$[Cu(phen)]^{2+}$	-	5.4 [1]
$[Cu(neo)]^{2+}$	-	3.6 [2]
$[Cu(tmp)]^{2+}$	-	2.2 [3]
$[Cu(batho)]^{2+}$	C0	1.3
[Cu(gly-val)(batho)]	C1	2.0
[Cu(gly-phe)(batho)]	C3	0.9
[Cu(ala-gly)(batho)]	C4	2.3
[Cu(ala-phe)(batho)]	C6	0.3
[Cu(phe-ala)(batho)]	C7	1.0

[Cu(phe-val)(batho)]	C7	0.8
[Cu(phe-phe)(batho)]	C7	1.0

Table S2. Aproximated DNA slope of the variation of the viscosity induced by the binding of the complexes

Complex	Slope
[$CuCl_2$(phen)]	0.24
[Cu(ala-phe)(phen)]	0.21
[Cu(ala-gly)(phen)]	0.24
phen	0.07
[$CuCl_2$(neo)]	0.13
[Cu(ala-phe)(neo)]	0.12
[Cu(ala-gly)(neo)]	0.14
neo	0.12
[$CuCl_2$(tmp)]	0.07
[Cu(ala-phe)(tmp)]	-0.03
[Cu(ala-gly)(tmp)]	0.10
tmp	0.12
[$CuCl_2$(batho)]	-0.28
[Cu(ala-phe)(batho)]	-0.29
[Cu(ala-gly)(batho)]	-0.24
batho	-0.36

Table S3. Selectivity index of the compounds (SI, IC_{50} on non tumor cells /IC_{50} on tumor cells of related origin)

Compound	SI (MCF-10/MDA-MB-231-)	SI (MCF-10/MCF-7)	SI (MRC-5/A549)
[Cu(gly-val)(batho)]	12.07	12.38	0.50
[Cu(gly-phe)(batho)]	4.55	3.77	0.97
[Cu(ala-gly)(batho)]	1.26	4.98	0.07
[Cu(ala-ala)(batho)]	7.66	2.48	0.68
[Cu(ala-phe)(batho)]	3.98	2.59	0.47
[Cu(phe-ala)(batho)]	2.25	3.36	0.76
[Cu(phe-val)(batho)]	5.77	2.44	0.24

[Cu(phe-phe)(batho)]	4.04	1.28	0.62
$[CuCl_2(batho)]$	5.85	1.00	0.98
Cisplatin	1.92	2.68	2.02

References

1. Iglesias, S., et al., *Synthesis, structural characterization and cytotoxic activity of ternary copper (II)–dipeptide–phenanthroline complexes. A step towards the development of new copper compounds for the treatment of cancer.* Journal of Inorganic Biochemistry, 2014. **139**: p. 117-123.
2. Alvarez, N., et al., *Synthesis and structural characterization of a series of ternary copper (II)-L-dipeptide-neocuproine complexes. Study of their cytotoxicity against cancer cells including MDA-MB-231, triple negative breast cancer cells.* Journal of Inorganic Biochemistry, 2020. **203**: p. 110930.
3. Alvarez, N., et al., *Tetramethyl-phenanthroline copper complexes in the development of drugs to treat cancer: synthesis, characterization and cytotoxicity studies of a series of copper(II)-l-dipeptide-3,4,7,8-tetramethyl-phenanthroline complexes.* JBIC Journal of Biological Inorganic Chemistry, 2022.
